# Global Asbestos Disaster

**DOI:** 10.3390/ijerph15051000

**Published:** 2018-05-16

**Authors:** Sugio Furuya, Odgerel Chimed-Ochir, Ken Takahashi, Annette David, Jukka Takala

**Affiliations:** 1 Japan Occupational Safety and Health Resource Center; Tokyo 136-0071, Japan; 2009aban@gmail.com; 2 Department of Environmental Epidemiology, Institute of Industrial Ecological Sciences, University of Occupational and Environmental Health, Kitakyushu 807-0804, Japan; odgerel@med.uoeh-u.ac.jp; 3 Asbestos Diseases Research Institute, University of Sydney, P.O. Box 3628, Rhodes, Sydney, NSW 2138, Australia; ken.takahashi@sydney.edu.au; 4 Health Partners, LLC, 125 Tun Jose Toves Way, Tamuning 96931, Guam; amdavid@guam.net; 5 WSH Institute, MOMSC, Singapore, ICOH c/o INAIL, Monteporzio Catone, 00078 Rome, Italy

**Keywords:** asbestos, ban, global estimates, costs

## Abstract

*Introduction*: Asbestos has been used for thousands of years but only at a large industrial scale for about 100–150 years. The first identified disease was asbestosis, a type of incurable pneumoconiosis caused by asbestos dust and fibres. The latest estimate of global number of asbestosis deaths from the Global Burden of Disease estimate 2016 is 3495. Asbestos-caused cancer was identified in the late 1930’s but despite today’s overwhelming evidence of the strong carcinogenicity of all asbestos types, including chrysotile, it is still widely used globally. Various estimates have been made over time including those of World Health Organization and International Labour Organization: 107,000–112,000 deaths. Present estimates are much higher. *Objective*: This article summarizes the special edition of this Journal related to asbestos and key aspects of the past and present of the asbestos problem globally. The objective is to collect and provide the latest evidence of the magnitude of asbestos-related diseases and to provide the present best data for revitalizing the International Labor Organization/World Health Organization Joint Program on Asbestos-related Diseases. *Methods*: Documentation on asbestos-related diseases, their recognition, reporting, compensation and prevention efforts were examined, in particular from the regulatory and prevention point of view. Estimated global numbers of incidence and mortality of asbestos-related diseases were examined. *Results*: Asbestos causes an estimated 255,000 deaths (243,223–260,029) annually according to latest knowledge, of which work-related exposures are responsible for 233,000 deaths (222,322–242,802). In the European Union, United States of America and in other high income economies (World Health Organization regional classification) the direct costs for sickness, early retirement and death, including production losses, have been estimated to be very high; in the Western European countries and European Union, and equivalent of 0.70% of the Gross Domestic Product or 114 × 10^9^ United States Dollars. Intangible costs could be much higher. When applying the Value of Statistical Life of 4 million EUR per cancer death used by the European Commission, we arrived at 410 × 10^9^ United States Dollars loss related to occupational cancer and 340 × 10^9^ related to asbestos exposure at work, while the human suffering and loss of life is impossible to quantify. The numbers and costs are increasing practically in every country and region in the world. Asbestos has been banned in 55 countries but is used widely today; some 2,030,000 tons consumed annually according to the latest available consumption data. Every 20 tons of asbestos produced and consumed kills a person somewhere in the world. Buying 1 kg of asbestos powder, e.g., in Asia, costs 0.38 United States Dollars, and 20 tons would cost in such retail market 7600 United States Dollars. *Conclusions*: Present efforts to eliminate this man-made problem, in fact an epidemiological disaster, and preventing exposures leading to it are insufficient in most countries in the world. Applying programs and policies, such as those for the elimination of all kind of asbestos use—that is banning of new asbestos use and tight control and management of existing structures containing asbestos—need revision and resources. The International Labor Organization/World Health Organization Joint Program for the Elimination of Asbestos-Related Diseases needs to be revitalized. Exposure limits do not protect properly against cancer but for asbestos removal and equivalent exposure elimination work, we propose a limit value of 1000 fibres/m^3^.

## 1. Introduction and Objective

The First Supplement to the “Occupation and Health—An Encyclopedia of Hygiene, Pathology and Social Welfare” of the International Labor Office, ILO, Vol. I, 999 pp.; Vol. II, 1310 pp. was published in 1938 and contained a Chapter on Asbestos [1,2]. It was the first time an International Labour Organization (ILO) document referred to cancer in relation to asbestos when identifying the criteria for action in asbestos-related workplaces. Asbestosis had been well recognized already at that time but the magnitude of the problem was revealed gradually when the carcinogenic properties of all kinds of asbestos fibres were convincingly identified.

It took 40 years to start international action. The ILO Asbestos Convention No. 162 was adopted in 1986 [3]. This Convention itself was a compromise of the pro-asbestos parties and those who wanted strict measures to stop using asbestos. Later, the Convention wording was misused against the original intent by using the expression “safe use of asbestos” out of context of ILO instruments.

Most of the asbestos exposures and adverse effects were considered to be linked to asbestosis and mesothelioma until recently—this millennium—when the magnitude of lung cancer, ovary and larynx cancer deaths have come to be better known. IARC—International Agency for Research on Cancer, of World Health Organization (WHO) has classified all types of asbestos causing these cancers and possibly other cancers and diseases. Other cancers may be confirmed as asbestos induced in future.

“ILO Resolution concerning asbestos in 2006” [4] finally corrected the missing parts of the Convention in stating that “all forms of asbestos, including chrysotile, are considered as known human carcinogens.” and “elimination of the future use of asbestos and the identification and proper management of asbestos currently in place are the most effective means to protect workers from asbestos exposure.”

Asbestos is the most significant factor for work-related cancer and work processes including asbestos that indirectly affects family members of asbestos workers, and the environment where where asbestos and related products are present. Lung cancer accounts for 54–75 per cent of all occupational cancer. Epidemiological studies indicate that occupational exposures cause 5.3–8.4 per cent of all cancers and among men, 17–29 per cent of all lung cancer deaths, according to best estimates. Asbestos accounts for 55–85 per cent of lung cancer and causes other cancers and other asbestos-related diseases (ARDs) today [5]. 

This paper summarizes the historical and related quantitative information of asbestos, key aspects of the past and present of the asbestos problem globally. The objectives of this paper are to collect and provide the latest evidence of the magnitude of asbestos-related diseases and to provide the present best data for revitalizing the ILO/WHO Joint Program on ARDs. Furthermore, the paper is intended to be an introduction to the special edition of papers on asbestos history in countries, regions and globally by IJERPH entitled “*Global Panorama of National Experiences in Public Health Actions to Ban Asbestos*”.

## 2. Materials and Methods

The main concept of the study design was to cover all ARDs by extending the scope of the paper of Odgerel C.-O. et al. [6]. This is done by using further data as explained below.

Materials and data were obtained from ILO Statistics [7] and Estimates, Work Safety and Health Institute, Singapore [8], WHO Mortality Database [9] the Institute of Health Metrics and Evaluation (IHME) [10] and national health statistics (Sources were provided where used). Crude mortality numbers and age-adjusted mortality rates were used to balance and compare outcomes for different countries and populations with varying age structures. Comparisons were made based on selected regions, countries, asbestos consumption, deaths and disability adjusted life years (DALY’s) for several confirmed ARDs, in particular, mesothelioma, lung cancer, larynx and ovary cancers, and asbestosis. In many workers’ compensation schemes, the individual worker’s smoking status is not a reason to exclude a victim from compensation.

Direct statistics and relatively reasonable estimates of asbestos related pneumoconiosis are available from WHO [9] and IHME statistics [10]. For pleural and peritoneal mesothelioma, which are caused by asbestos exposure, this is also the case. However, a serious under-diagnosing or non-diagnosing is a source of error for recording, in particular, lung cancer cases. Recorded lung cancer, ovarian and larynx cancers do not usually indicate asbestos as a cause of death. Diagnosing these properly for an individual needs a post mortem including fibre counts of the lung tissue, which is not a usual practice for the huge majority of cases. Consequently, the numbers may be estimated either using the attributable fraction method based on quantity of asbestos exposure and number of exposed workers. Furthermore, the number of mesothelioma cases where almost all cases/deaths are linked to asbestos exposure can be used as a proxy for asbestos exposure exactly in the same way as has been done for the IHME estimates on the Global Burden of Diseases and Injury GBD 2016 [10]. 

Statistics related to ARDs were shown by comparing the most recently available sources [5,6,7,8,9,10,11,12,13]. The mid-point of the highest and lowest results was obtained, and selected geographical estimates on ARDs were provided. Details of estimation are provided in the results section. 

Cost comparisons were made using the estimated disability adjusted life years (DALYs) caused by asbestos as compared to an ideal case where no ARDs, no asbestos consumption and no asbestos exposures were present [10]. This itself is somewhat challenging and may cause a source of error as it is practically impossible to find a populated location without any asbestos fibres in air globally. Statistics are not available from most countries in the world, and proxy estimates based on comparable countries and regions were made when no data were available. Exposures and negative outcomes were estimated separately when data were available for occupational exposures and non-occupational sources. The IHME/GBD number of DALYs, Years of Lost Life (YLL) and Years Lived in Disability (YLD) were taken as a baseline, while the mortality numbers were extrapolated from available data by Odgerel C.-O. et al. [6]. The method is equivalent to that of an ILO study on economic costs of occupational injuries and illnesses, including cancer. That was based on results of a team of researchers including ILO, International Commission on Occupational Health, Work Safety and Health Institute of Singapore, Finnish Institute of Occupational Health, ministries of Finland and Singapore and the European Agency for Safety and Health at Work [7]. 

## 3. Results

A summary of present knowledge related to mesothelioma is presented in Table 1. Rushton et al. previously reported the occupational component of work-related mesothelioma to be 94.9% [14]; however, we used the percentage of 91.4 (Please see Supplementary Table S1) to calculate the work related mesothelioma. 

Mesothelioma deaths were estimated by GBD 2016 to be 30,208. Please refer to Supplementary Table S1 for details. The latest scientific paper by Odgerel C.-O. et al. [6] estimated the number of deaths to be 38,388 using the asbestos consumption-adjusted method. Equivalent work-related outcomes were correspondingly 27,612 (GBD 2016) and 35,087 (Odgerel C.-O. 2017). Earlier data for GBD 2016 are given as a comparison for China and EU28 in Table 1. Further details and tables on country level mesothelioma deaths by Odgerel C.-O. et al. [6] are given by the authors; in addition, a mesothelioma excel table by country based on WHO data is included in the CEJOEM journal web version [11].

**Figure 1:**
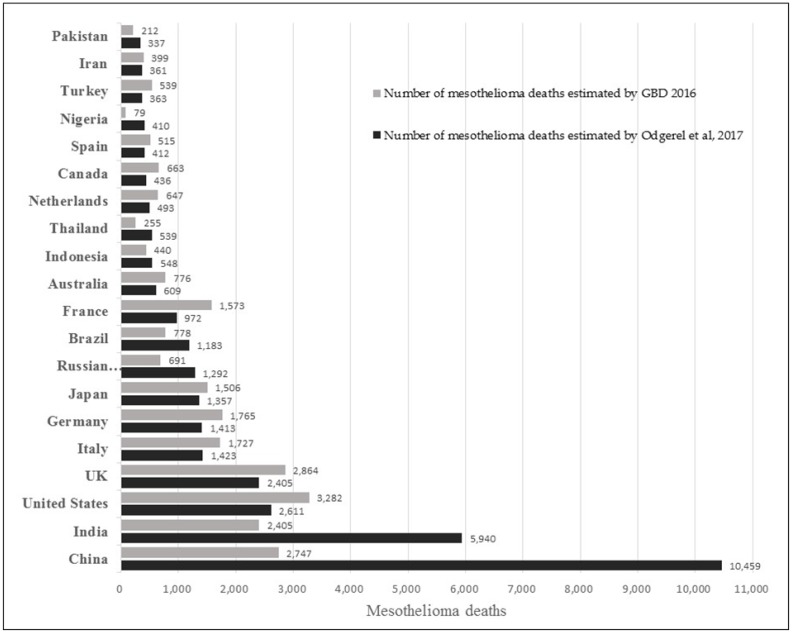
Comparison of Global Burden of Mesothelioma Deaths for leading countries in terms of mesothelioma deaths [6,10].

Supplementary Table S2 and Figure 1 compare the outcomes of the two estimation methods for mesothelioma of the highest asbestos using countries in the past, as the mesothelioma case number is a reasonable proxy for asbestos exposures and is interlinked with other asbestos-caused cancers. Asbestosis victims may develop cancers as well while cancer and asbestosis do not necessarily develop simultaneously. 

**Figure 2:**
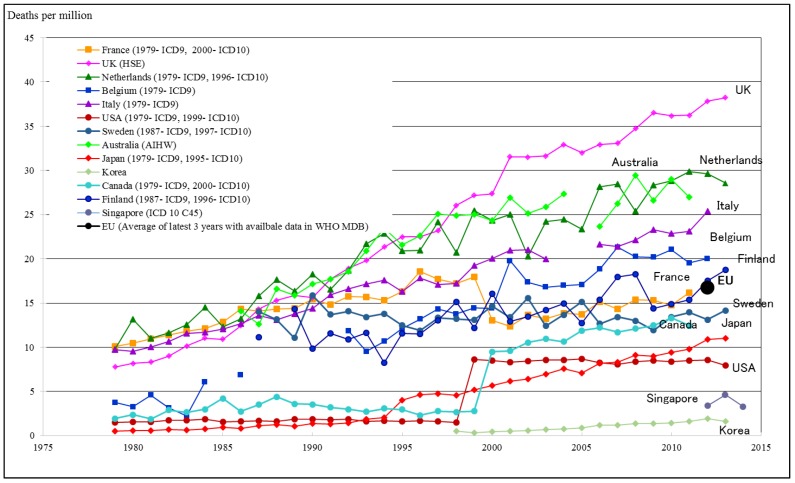
Mesothelioma death rate for selected countries. WHO Mortality Database, ICD 10: C45 Mesothelioma, ICD 9: 163 Malignant Neoplasm of Pleura; UK: Health and Safety Executive Statistics—Mesothelioma, http://www.hse.gov.uk/statistics/causdis/meso.htm; Australia: National Cancer Statistics Clearing House of Australian Institute of Health and Welfare (AIHW).

Evidence on the rising numbers of numbers of mesothelioma deaths is given in Figure 2. It appears that these numbers increase for some time in future [15]. Figure 2 shows that there is so far no solid evidence that the total mesothelioma numbers would be starting to go significantly down in any country while reports of younger cohorts in Sweden—and the Netherlands—show that the cessation to use asbestos in the 1980’s started to have impact after the long latency period [16]. 

**Figure 3:**
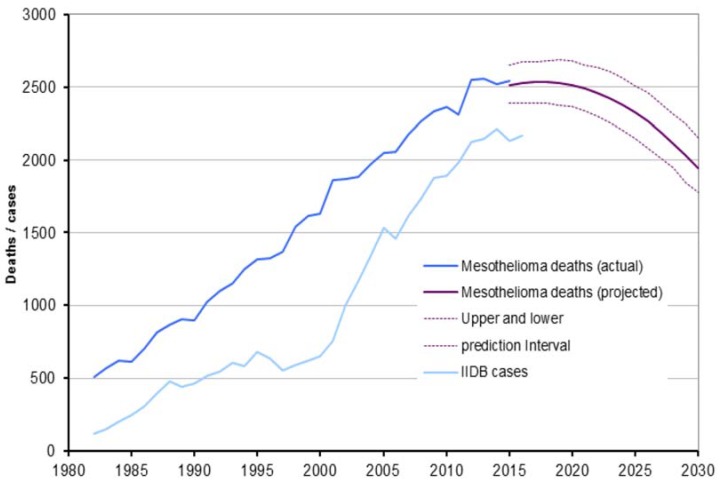
Mesothelioma annual deaths, Industrial Injuries Disablement Benefit (IIDB) cases and projected future deaths to 2030 in GB, Health and Safety Executive/UK 2016.

Supplementary Table S1 shows the relative importance of lung cancer in the burden caused by asbestos in major asbestos-using countries and globally. The data are based on the relatively conservative estimates of the GBD 2016 outcome of the IHME. The evidence from the United Kingdom (UK) shows that the numbers are likely to start going down in the 2020’s based on the gradually reduced asbestos consumption and exposures first and later legally banning the use altogether, see Figure 3 [13]. In addition to numbers of deaths, data exist on rates per 1 million population.

**Figure 4:**
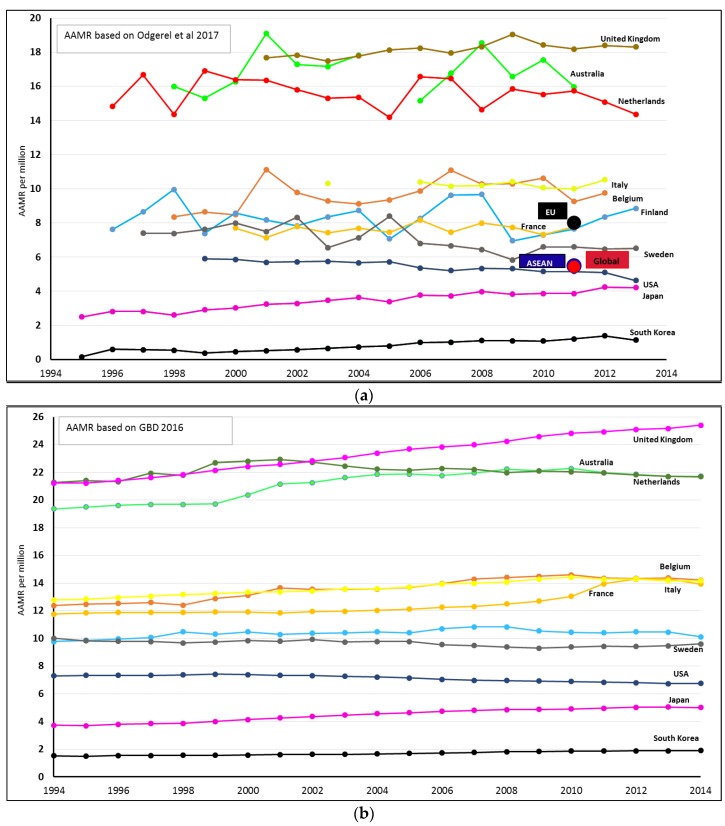
Age-adjusted mortality rate of mesothelioma for selected countries based on Odgerel C-O 2017 [6] (**a**); GBD 2016 study [10] (**b**).

Figure 4 shows the age-adjusted rates for selected countries, and an increasing trend is visible in some countries in the GBD 2016 [10] measures while the trends based on reported data in Odgerel C.-O. [6] paper are less clear. This may be caused by the recent increases of young migrant populations in selected countries that may result in significantly larger young populations today as compared to the originally exposed populations 30–50 years earlier. Note that in the Odgerel C.-O. [6] paper, for each calendar year, age-adjusted rates were directly calculated from actually reported numbers of mesothelioma deaths in these countries, without accounting for statistical fluctuation caused by generally low rates. In contrast, the GBD 2016 [10] data provide estimates accounting for fluctuation of “rare events.” It is most likely that the estimates in the GBD 2016 data [10] “smooth out” fluctuations by statistical modelling.

Based on these numbers and the global peak annual consumption of asbestos globally in 1980, which was 4,728,619 metric tons [17] and the Table 2 number in 2016 of asbestos-caused deaths by the mid-point value of asbestos deaths, 255,000 deaths would provide a rough estimate of 18.4 tons of asbestos consumption killing one person 37 years later. Using the lowest GBD2016 [10] estimate provides an amount of 19.4 tons causing one death. It would be safe to say that—as a rule of thumb—20 tons of asbestos use will cause one death. 

It appears that the mesothelioma death numbers are consistently increasing. This leads to the conclusion, based on the earlier method of using the mesothelioma deaths as a proxy of asbestos exposure, that asbestos-related cancer deaths are also increasing. The peak consumption of asbestos in 1980 causes one death through the use and consumption of slightly less than 20 tons. The increasing mesothelioma and other cancer numbers would mean that the number of all asbestos-caused deaths are expected to increase in future.

**Figure 5:**
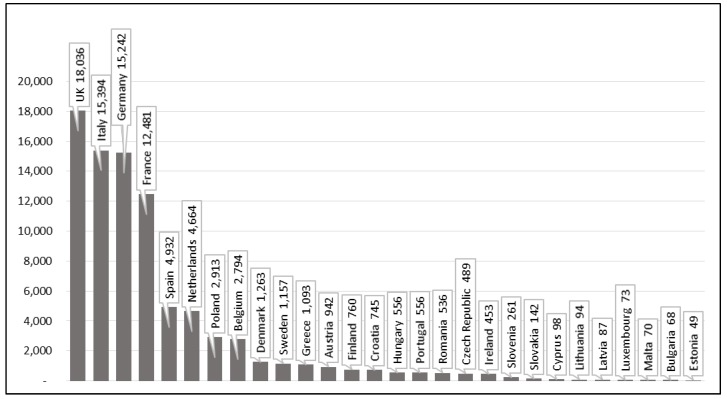
Asbestos cancer deaths at work in EU28 [6].

The EU28 (28 countries of European Union) are one of the heaviest exposed world region, and Figure 5 provides details of the GBD 2016 estimates [10].

The ILO and the European Agency for Safety and Health at Work have estimated the costs of poor safety and health at work. The overall global estimate was equal to 3.94% of the global Gross Domestic Product (GDP), equaling 2,966,000 million USD. This estimate was made using the work-related DALYs as a share of a maximum number of years of gainfully productive worker years if no one was out of work due to occupational injuries and work-related diseases [18]. Asbestos is likely to be the most significant individual occupational risk factor and consequently, the most significant component in such economic losses. Using the same method for specific countries and regions, one may estimate the losses caused by asbestos-related risks.

Based on the GBD 2016 [6] estimated 85,419 work-related deaths and 1277 million DALYs [9] in the European Union of 218.3 million workers and an equal number of productive years, the rate: DALY asbestos/Employment years without losses will result in 0.70% loss of productive output caused by asbestos at work, which could be compared to the GDP of the region.

The United States of America (USA) has a slightly lower incidence rate, lower loss rate and smaller population but higher GDP per capita. Compared to the EU, where asbestos is estimated to cause productivity losses at about 0.70% of GDP, amounting to 114,900 million USD, the USA has asbestos-related productivity losses of approximately 0.36% of GDP, or 86,100 million USD losses caused by asbestos.

All WHO region “High income countries” together had an estimated loss of 0.48% of GDP caused by asbestos risk, while the global rate and losses are significantly lower due to lower asbestos use—so far in the past—and much lower average GDP levels. Comparing countries at different levels of development globally or regionally based on different GDP levels may not be appropriate.

These estimates were based on lower mesothelioma estimates. Using the latest and higher mesothelioma estimate (38,400) as a proxy for asbestos exposure, all these numbers, rates and costs will be higher; see Table 1 and Table 2. As a result, asbestos-related lung cancer, other cancers and asbestosis death numbers of the high end of the estimate (260,029) would result in a corresponding higher DALY level and higher cost estimate. 

The calculation of all occupational cancer costs of 104,000 deaths (disability costs not included) in the EU28 multiplied by the Virtual Statistical Life (VSL = 4 million EUR per cancer death) used by the European Commission would result in 416 × 10^9^ USD for occupational cancer, and 340 × 10^9^ related to asbestos exposure at work in EU28, These costs for the European Union, 85,419 deaths, are much higher than the traditional estimates presented here above. 

A detailed study by Health and Safety Executive in the UK on occupational cancer—of which asbestos was the overwhelming main cause, yielded a cost in the UK of 12.3 × 10^9^ GBP in 2010 [18]. Lung cancer (£6.8 billion) and mesothelioma (£3.0 billion were the main causes of costs. The method used above for economic costs of occupational cancer and asbestos-related cancer yielded 18.3 × 10^9^ USD in 2015, which is practically equal to the result of a detailed study in the UK according to the GBP/USD rate and the different years [19]. 

## 4. Discussion

The metrics to appropriately estimate the magnitude of asbestos-related disorders are gradually improving, and the size of the problem is increasing. Meanwhile “fake news” not based on facts is still actively advocated against all and overwhelming scientific evidence of the carcinogenicity of all types of asbestos. 

In practice, however, most asbestos-caused cancers are not reported, recorded and compensated for and in most countries, and none of them are properly identified and compensated. The synergistic additive or sometimes multiplicative impact of smoking and asbestos often confuses and masks the identification of asbestos-caused problems. Major ARDs and, in particular, lung cancer, are typical major manifestations of multiple simultaneous exposures complicating individual diagnosis. Depending on the reliability of source materials and methods of estimation, a considerable number of asbestos exposure victims may have been be classified as victims of smoking, thus producing gross under-estimates of the role of asbestos. According to the definition of the attributable fraction, the baseline for estimation should be to count the difference between the numbers of negative outcomes in studied comparable populations when the related exposure, such as asbestos exposure, is or is not present. This means independence of the impact of other simultaneously present factors. The practice of adjusting attributable fractions for smoking may not be ethically sound.

It is not an easy task to estimate the intangible costs of using asbestos. Production losses are simpler to calculate. While the methods were quite different, the background information of the magnitude and the numbers of deaths are obviously based on the same sources and research reports [9]. 

The numbers and costs are increasing practically in every country and region in the world. Asbestos has been banned in 55 countries but is used widely today: 2,030,000 tons consumed annually according to latest available consumption data. Every 20 tons of asbestos produced and consumed kills a person somewhere in the world. Buying 1 kg of asbestos in powder format, e.g., in Asia, costs 0.38 USD and 20 tons would cost 7600 USD. The present asbestos consumption and exposure will cause negative outcomes 30–50 years later.

However, when applying the Value of Statistical Life of 4 million EUR per cancer death used by the European Commission we could arrive at a much higher cost, while the human suffering and loss of life is impossible to quantify. Furthermore, the same Value of Statistical Life case cost could be applied to the global deaths (222,000 Table 2) if based on the conservative GBD 2016 occupational risks estimate, or the alternative latest estimate of 260,029 in Table 2. This would go far beyond the practice of just looking at the productive losses through DALYs. The earlier presented DALY and loss of productivity based estimates are, however, in line with the ILO’s costs estimate method. 

While banning asbestos is a simple way to stop future exposures, the management of existing asbestos in buildings and structures and the work to remove of asbestos needs exposure limits. The present limits are not protective enough, and millions of workers and others are still exposed in countries that banned asbestos tens of years ago. A present commonly used limit of 0.1 fibres/cm^3^ means 100,000 asbestos fibres in one cubic meter m^3^. Human lungs will easily inhale 100,000 asbestos fibres in an hour. An appropriate limit at work would be 1000 fibres/m^3^. The proposed value is a simplified exposure limit and is based on the Dutch Expert Committee that suggested three values for work exposures: 2000 fibres for chrysotile, 1300 for mixed fibres, and 420 fibres/m^3^ for amphibole fibres [20], and slightly lower for non-occupational exposure. 

## 5. Conclusions

Present efforts to eliminate this man-made problem and exposure leading to the present epidemiological disaster have been insufficient in most countries in the world. Applying programmes and policies, such as the elimination of all kinds of asbestos use—that is banning of new asbestos use and tight control and management of existing structures containing asbestos—need strengthening and follow up. The present policies and practices need revision and resources. The ILO/WHO Joint Programme for the Elimination of Asbestos-related Diseases needs to be revitalized. Exposure limits do not protect properly against cancer, but for asbestos removal and equivalent exposure elimination work we propose a limit value of 1000 fibres/m^3^.

## Figures and Tables

**Table 1:** Summary of most recent information related to mesothelioma.

Sources		Global	China	EU28
Takala 2015 [5]				10,368
Odgerel C.-O. 2017 [6]	Reported	15,011	NA ***	8363
	Estimated *	21,247–23,377	6456–10,459	NA ***
	Total	36,258–38,388		
Global Burden of Diseases 2016 [10]		30,208	2747	10,700
Work-related mesothelioma	GBD 2016 [10]	27,612		
	Odgerel C.-O. 2017 [6]	35,087 **		

* Mesothelioma deaths were estimated based on continental region, % of employment in industrial sector and asbestos consumption. Please refer to the original article for details; ** Calculated from asbestos adjusted estimation which is 38,388. *** China didn’t report mesothelioma data to WHO, thus, it’s estimated; and all countries in EU reported the data to WHO, thus, the estimation was unnecessary.

**Table 2:** Asbestos-related lung cancer and other asbestos-related deaths.

Methods of Estimated Lung Cancer Deaths Using Mesothelioma as a Proxy for Asbestos Use	Lung Cancer or Asbestos Related Cancers/Mesothelioma Death Ratio	Global	China	EU28
McCormack, Peto et al. (2013) average estimate [12] using chrysotile, lung cancer, all exposed ^i^, GBD 2016	6.1	184,269		
McCormack, Peto et al. (2013), low–high estimates, lung cancer, all exposed ^ii^, GBD 2016	2.0–10	55,224–302,208		
Asbestos-related cancers ^iii^ (occupational exposure to asbestos, IHME 2016)	6.92 ^iv^			
Mesothelioma, work		27,612	2178	10,480
ARLC (Asbestos related lung cancer), work		181,450	17,971	70,291
Ovarian cancer, work		6022	270	2868
Larynx cancer, work		3743	198	1287
Total asbestos related cancer at work (GBD 2016/Odgerel C-O 2017)		22,322_work_/242,802_work_^v^		
		Mid point 232,562		
Total asbestos (all exposed) using mesothelioma _all-work_ as proxies (GBD 2016/Odgerel C-O 2017)		243,223_all_/260,029_all_^vi^		
		Mid point 254,626		

^i,ii^—included all exposed cases (occupational + non-occupational). ^iii^—Mesothelioma, ARLC, ovarian and larynx cancer. ^iv^— (ARLC + Ovarian + Larynx cancer + /Occupational mesothelioma. ^v^—35,087 × 6.92. ^vi^—38,388 × 6.92.

## Supplementary Materials

The following are available online at http://www.mdpi.com/1660-4601/15/5/1000/s1, Table S1: All estimated asbestos-related deaths at work (semi-occupational and non-occupational), Table S2: Comparison of Global Burden of Mesothelioma Deaths (Odgerel C.-O. et al. 2017/GBD 2016).
