# Supplementary material for: Global Asbestos Disaster
Source: Int J Environ Res Public Health. 2018 May 16;15(5):1000. doi: 10.3390/ijerph15051000 (PMC5982039; doi:10.3390/ijerph15051000)
Supplement: Supplementary file 1 [file ijerph-15-01000-s001.zip › 180516_Supplementary tables.docx]

Supplementary Table 1. All estimated asbestos related deaths (semi-occupational and non-occupational)

Supplementary table 2. Comparison of Global Burden of Mesothelioma Deaths (Odgerel et al 2017/ GBD 2016)

GBD2016: GBD Compare database: https://vizhub.healthdata.org/gbd-compare/ (accessed 02/02/2018)

Estimation by Odgerel et al: Chimed-Ochir Odgerel, Ken Takahashi, et al. Estimation of the global burden of mesothelioma deaths from incomplete national mortality data Occup Environ Med 2017;0:1–8. doi:10.1136/oemed-2017-104298: <http://oem.bmj.com/content/early/2017/09/02/oemed-2017-104298>

Supplementary file S3: <http://oem.bmj.com/content/early/2017/09/02/oemed-2017-104298#DC1>

*: Countries reported mesothelioma death to WHO.
